# A Ten-Year Retrospective Cohort Study on Neck Collar Immobilization in Trauma Patients with Head and Neck Injuries

**DOI:** 10.3390/medicina59111974

**Published:** 2023-11-09

**Authors:** Shu-Jui Lee, Lin Jian, Chi-Yuan Liu, I-Shiang Tzeng, Da-Sen Chien, Yueh-Tseng Hou, Po-Chen Lin, Yu-Long Chen, Meng-Yu Wu, Giou-Teng Yiang

**Affiliations:** 1 Department of Emergency Medicine, Taipei Tzu Chi Hospital, Buddhist Tzu Chi Medical Foundation, New Taipei 231, Taiwan; 2 Department of Emergency Medicine, School of Medicine, Tzu Chi University, Hualien 970, Taiwan; 3 Department of Medical Education, Changhua Christian Hospital, Changhua 500, Taiwan; 4 Department of Medicine, College of Medicine, Tzu Chi University, Hualien 970, Taiwan; 5 Department of Orthopedic Surgery, Taipei Tzu Chi Hospital, Buddhist Tzu Chi Medical Foundation, New Taipei 231, Taiwan; 6 Department of Orthopedics, School of Medicine, Tzu Chi University, Hualien 970, Taiwan; 7 Department of Research, Taipei Tzu Chi Hospital, Buddhist Tzu Chi Medical Foundation, New Taipei 231, Taiwan

**Keywords:** head and neck injury, c-spinal immobilization, neck collar, mortality

## Abstract

*Background and Objectives*: In the context of prehospital care, spinal immobilization is commonly employed to maintain cervical stability in head and neck injury patients. However, its use in cases of unclear consciousness or major trauma patients is often precautionary, pending the exclusion of unstable spinal injuries through appropriate diagnostic imaging. The impact of prehospital C-spinal immobilization in these specific patient populations remains uncertain. *Materials and Methods*: We conducted a retrospective cohort study at Taipei Tzu Chi Hospital from January 2009 to May 2019, focusing on trauma patients suspected of head and neck injuries. The primary outcome assessed was in-hospital mortality. We employed multivariable logistic regression to investigate the relationship between prehospital C-spine immobilization and outcomes, while adjusting for various factors such as age, gender, type of traumatic brain injury, Injury Severity Score (ISS), Revised Trauma Score (RTS), and activation of trauma team. *Results*: Our analysis encompassed 2733 patients. Among these, patients in the unclear consciousness group (GCS ≤ 8) who underwent C-spine immobilization exhibited a higher mortality rate than those without immobilization. However, there was no statistically significant difference in mortality among patients with alert consciousness (GCS > 8). Multivariable logistic regression analysis revealed that advanced age (age ≥ 65), unclear consciousness (GCS ≤ 8), major traumatic injuries (ISS ≥ 16 and RTS ≤ 7), and the use of neck collars for immobilization (adjusted OR: 1.850, 95% CI: 1.240–2.760, *p* = 0.003) were significantly associated with an increased risk of mortality. Subgroup analysis indicated that C-spine immobilization was significantly linked to an elevated risk of mortality in older adults (age ≥ 65), patients with unclear consciousness (GCS ≤ 8), those with major traumatic injuries (ISS ≥ 16 and RTS ≤ 7), and individuals in shock (shock index > 1). *Conclusions*: While our findings do not advocate for the complete abandonment of neck collars in all suspected head and neck injury patients, our study suggests that prehospital cervical and spinal immobilization should be applied more selectively in certain head and neck injury populations. This approach is particularly relevant for older individuals (age ≥ 65), those with unclear consciousness (GCS ≤ 8), individuals experiencing major traumatic injuries (ISS ≥ 16 or RTS ≤ 7), and patients in a state of shock (shock index ≥ 1). Our study employs a retrospective cohort design, which may introduce selection bias. Therefore, in the future, there is a need for confirmation of our results through a two-arm randomized controlled trial (RCT) arises, as this design is considered ideal for addressing this issue.

## 1. Introduction

Over 50 million patients visit emergency departments for trauma-related reasons each year, which increases burden of health care costs [1,2]. Head and neck injuries (HNI) account for 39% of all injury casualties [3]. Injury severity can range from soft tissue lacerations to traumatic neurological injury. The mechanism may be broad from minor falls to severe motor vehicle accidents [4]. Although the prevalence of concomitant cervical spinal injury in patients with TBI was 6.5%, prehospital spinal immobilization is widely used for cervical in-line stabilization in highly suspect C-spinal injury patients [5]. Current practices are based on the assumption that head and neck injury may cause neurological injury due to an unstable spinal column without immobilization. However, C-spinal immobilization entails its own risks of complications and possible adverse effects, including increased risk of respiratory compromise [6], back and neck pain [7,8,9], pressure sores [10], and increased intracranial pressure [11]. C-spinal immobilization may also prolong rescue airway management and on-scene time to delay transport [12,13,14]. In addition, airway compromise and elevation of intracranial pressure may also trigger hypoxemia, intracranial hypertension, and hypoperfusion in severe TBI. More and more studies recommend that the HNI population with clear consciousness should early receive spinal clearance at the scene based on National Emergency X-Radiography Utilization Study (NEXUS) criteria or clinical symptoms.

In the consciousness-unclear population or severe injury mechanism, especially in the setting of a motor vehicle collision, fall, or sports-related injury, prophylactic spinal immobilization in the traumatic injury group is usually used due to difficult evaluation until exclusion of unstable spinal injury or appropriate diagnostic imaging. But the effect of prehospital C-spinal immobilization in these populations remains unclear. In Wesley B. Vanderlan et al. [15], the authors revealed that penetrating neck trauma with C-spine immobilization was associated with a high risk of mortality and an increased risk of cardiopulmonary resuscitation (CPR). In Elliott R. Haut et al. [16], the authors showed similar result in spine-immobilized penetrating trauma patients. Therefore, the benefit of prehospital C-spine immobilization has been called into question, because clinical benefit may not be worth delaying definitive care [17]. In the blunt HNI population and shock condition, there is a lack of high-level evidence on the effect of prehospital cervical spine immobilization on head and neck injury patient outcomes [18]. In our study, we aim to determine the impact of prehospital c-spinal immobilization in suspected HNI patient outcomes, whether removing a neck collar can provide benefit in suspected HNI, and which subgroups have less benefit from C-spine immobilization. Additionally, we also tried to investigate potential factors that may influence the benefit of C-spine immobilization. We believed prehospital c-spinal immobilization should be more specific and selective in population.

## 2. Methods

### 2.1. Study Setting and Patients Data Source

We conducted a retrospective cohort study of trauma patients in Taipei Tzu Chi Hospital from January 2009 to May 2019 by Taipei Tzu Chi Hospital, Buddhist Tzu Chi Medical Foundation, New Taipei City. The Institutional Review Board of Taipei Tzu Chi Hospital gave approval for this study (IRB number: 11-XD-148). Patient data were retrospectively reviewed from the Taipei Tzu Chi Hospital Trauma Database. Patients were included if they visited Taipei Tzu Chi Hospital and had hospitalization history from January 2009 to May 2019. These patients received outpatient department following up. The exclusion criteria for this study cohort were missing data on important parameters including in-hospital data and clinical outcome.

The detailed demographic, overall survival and clinical outcome data were collected from the trauma database, computerized records, and charts. The basic characteristics of the patients included age and sex, comorbid conditions, injury location, types of injuries, and EMT treatment. The in-hospital parameters included triage, activation of trauma team, in-hospital vital sign, and clinical outcome. Patients were divided into non-geriatric patients and geriatric patients by a cut-off value of 65 in age for the subgroup analysis [19]. In prehospital management, rescue airway included supraglottic airway or endotracheal tube intubation. Oxygen support included nasal cannula, oxygen mask, and non-rebreathing oxygen mask. Injury severity was analyzed by Injury Severity Score (ISS), Revised Trauma Score (RTS), New Trauma and Injury Severity Score (TRISS) and New Injury Severity Score (NISS). We adopted two major score, ISS and RTS, as the indices of trauma severity. ISS was calculated by summing the square of the 3 highest Abbreviated Injury Scale scores of different injury body regions. RTS was calculated by the following formula: RTS = (GCS score coded × 0.9368) + (SBP coded × 0.7326) + (RR coded × 0.2908). We dichotomized major trauma by ISS with cut-off value of 16 and RTS with a cut-off value of 7 [20,21]. We also used the shock index, defined as the heart rate (HR) divided by systolic blood pressure (SBP), to dichotomize shock status by a cut-off value of 1 [22]. The clinical outcome was analyzed via hospitalization time, ICU admission, re-admission ICU, ICU admission time, operation, re-operation and mortality.

### 2.2. Statistical Analysis

All continuous data were analyzed as normally distributed by the Kolmogorov–Smirnov test. Dichotomous and categorical variables are reported as sample numbers (percentages). Continuous variables are reported as the median (interquartile range or Q1–Q3). Non-parametric ANOVA or the Mann–Whitney U test was used for comparison of continuous variables. The Pearson chi-squared test or Fisher’s exact test was used for comparison of categorical and nominal variables. Multivariable logistic regression was used to determine the association between parameters and clinical outcomes in the head and neck injury population. Variables that had *p* < 0.10 on the chi-squared test or the Mann–Whitney U test were selected for multivariable logistic regression analysis using the forced entry method. Variance inflation factors were used to recognized multicollinearity among a set of explanatory variables. The VIFs are less than 10, indicating that the multicollinearity does not pose a serious problem for those models, and the VIFs exceeding 10 are signs of serious multicollinearity requiring correction. We drop the variables with VIFs exceeding 10 to eliminate the extreme multicollinearity. The model fit was assessed using the Hosmer–Lemeshow goodness-of-fit test. The discrimination of the multivariable regression model was tested using the area under the receiver operating characteristic curve (AUROC) for mortality outcome. In the subgroup analysis, multivariable logistic regression was used via SPSS software (Version 13.0 SPSS Inc., Chicago, IL, USA) for statistical analysis. Statistically significance was defined as *p*-value < 0.05.

## 3. Results

### Patient Characteristics and Prehospital Analysis

**Figure 1:**
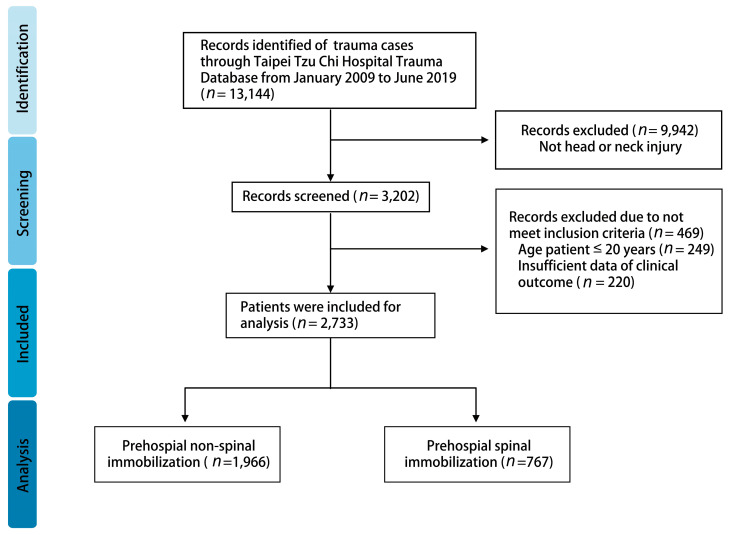
Flow diagram of included patients.

A total of 13,144 patients were eligible for review in the Taipei Tzu Chi Hospital Trauma Database. After exclusion of patients without head and neck injury (*n* = 9942), 3202 patients remained. Among them, 469 patients were excluded for missing data or age below 20 years. The remaining 2733 patients were included in this study (Figure 1). The characteristics of the total included patients were shown in Table 1. There were 2733 patients included with median age (IQR): 62 (45–77) and 1632 (59.7%) patients were male. In total, 1245 (45.6%) patients were age ≥65 and 1488 (54.4%) patients were age < 65. The Glasgow coma scale (GCS) at triage in the C-spine immobilization group (CSI) is lower than in the non-C-spine immobilization group (nCSI); and in the CSI group, there are up to 227 (29.6%) patients with GCS ≤ 8. In the CSI group, triage level I accounted for approximately 42.8%, which was more than in the nCSI group (14.0%) and the proportion who required activation of trauma team is higher in the CSI group [259 (33.8%) vs. 66 (3.4%), *p* < 0.001]. Isolated TBI accounted for approximately 65.3% in all patients and more in the CSI group [1396 (71.0%) vs. 388 (50.6%), *p* < 0.001]. The proportion requiring EMT management was higher in the CSI than in the nCSI population, including stopping bleeding and banding, spinal board immobilization, splint immobilization, oxygen support, rescue airway management, and cardiopulmonary resuscitation (CPR). In the analysis of where accidents occurred, most occurred on the street, 52.8%, with a higher percentage, 69.8%, in the CSI group. Motor vehicle collision is the major injury type in the total head and neck injury population (34.1%), followed by contusion (34.1%). In the nCSI group, contusion is the major injury type. The injury severity analysis revealed that the CSI group had more severe injuries than the nCSI group, from RTS, ISS, NISS, and TRISS. There was also a higher major traumatic population in the CSI group than in the nCSI group based on ISS ≥ 16 [445 (22.6%) vs. 379 (49.4%), *p* < 0.001] and RTS ≤ 7 [273 (13.9%) vs. 328 (42.8%), *p* < 0.001]. In the clinical outcome analysis, the CSI group had a higher proportion of ICU admissions, re-ICU admissions, operations, re-operations, complications, and death. Length of stay (LOS) in ICU was also longer in the CSI population but total LOS was not significantly different between the two groups.

The subgroup analysis of consciousness-alert (GCS > 8) and -unclear population (GCS ≤ 8) was shown in Table 2. Age < 65 and isolated TBI populations were the major populations in the CSI group with GCS > 8 or GCS ≤ 8. Based on injury severity of ISS and RTS, the major traumatic population was also higher in the CSI group than in the nCSI group in both the consciousness-alert (GCS > 8) and -unclear population (GCS ≤ 8) groups.

The unclear population (GCS ≤ 8) with the C-spine immobilization group had higher mortality rates than the nCSI group but there was no significant result in the consciousness-alert group (GCS > 8). In the isolated TBI population with or without consciousness-clear, the mortality rate is not significantly different between nCSI and CSI (Table 3). However, interestingly, a mixed TBI population with consciousness-unclear (GCS ≤ 8) had higher mortality rates in the C-spine immobilization group than the non-immobilization group [nCSI: 19 (40.4%) vs. CSI: 102 (78.5%), *p* < 0.001].

Multivariable logistic regression of in-hospital mortality revealed that old age (age ≥ 65), consciousness-unclear (GCS ≤ 8), and major traumatic injury (ISS ≥ 16 and RTS ≤ 7) were significantly associated with an increased risk of mortality. Male, TBI type, injury type and activation of trauma team were not at a statistically significant level. Neck collar immobilization in the head and neck injury population was significantly associated with an increased odds of mortality (adjusted OR: 1.850, 95% CI: 1.240–2.760, *p* = 0.003) (Table 4). The Hosmer–Lemeshow test showed adequate fit (χ^2^ = 10.60, *p* = 0.226) and the AUROC of the multiple logistic regression model for association of neck collar immobilization and mortality was 0.921 with 95% CI: 0.905–0.937. In the subgroup analysis (Table 5), compared to the nCSI group, the c-spine immobilization group had a higher odds ratio of mortality in old age (age ≥ 65), consciousness-unclear (GCS ≤ 8), major traumatic injury (ISS ≥ 16 and RTS ≤ 7), mixed TBI, isolated TBI, and shock population.

## 4. Discussion

The present study explored the association between prehospital c-spinal immobilization and mortality in the HNI population and observed that the C-spinal immobilization group has a higher risk of mortality than the non-C-spinal immobilization group (aOR: 1.850, 95% CI: 1.240–2.760, *p* = 0.003). In the HNI population with old age (age ≥ 65), consciousness-unclear (GCS ≤ 8), major traumatic injury (ISS ≥ 16 or RTS ≤ 7), and shock status (shock index < 1), the C-spinal immobilization group has a higher adjusted OR of mortality than the non-C-spinal immobilization group.

This study has some strengths. First, our study involved the Asian population, which has not been widely investigated in previous studies on the association of prehospital c-spinal immobilization and mortality in the HNI population. Second, our study included many confounders in the multivariable logistic regressions, such as injury mechanism and TBI type, and different injury severity indices, and prehospital management. Previous studies did not include these clinical variables due to limitations of the database. Third, our study used the head and neck injury population to investigate neck collar effects instead of the definite diagnosis, as a prehospital setting for EMS and useful guide for emergency physicians. Finally, our results showed the association between prehospital C-spinal immobilization and mortality in the HNI population. In addition, we highlighted that the benefits of prehospital C-spinal immobilization may be less in HNI patients with old age (age ≥ 65), consciousness-unclear (GCS ≤ 8), major traumatic injury (ISS ≥ 16 or RTS ≤ 7), and shock status (shock index < 1).

Prehospital C-spinal immobilization is usually used to prevent neurologic complication in HNI. Overimmobilization may occur in difficult-to-manage patients, such as the old age, consciousness-unclear, shock and severe injury mechanism populations. However, in Mark Hauswald et al. [23], a 5-year retrospective chart review study, the authors found that prehospital immobilization in blunt spinal injuries has little or no effect on neurologic outcome, with an adjusted OR of 2.03 of disability under 95% CI 1.03–3.99. In terms of physical and biomechanical reasons, energy deposition during an injury is a complex process and spinal injury at the scene is usually caused by ejection from vehicles contacting the vehicle structure or the ground. Compared to a direct event, the energy of the spine’s normal motion due to no immobilization is low, which may explain why immobilization immediately after injury has little effect [23]. Another study, conducted in Taiwan, with lightweight motorcycle injuries showed that 63/8633 (0.73%) patients had cervical spine injury and only 16 patients received surgical intervention [24]. There was no significant correlation between cervical spine injury and neck collar whether applied or not [24]. In addition, the authors have reported that there was a significant correlation of supraclavicular lesion, neck pain and neurologic deficit in patients with c-collars. Some studies also mentioned that c-spinal immobilization carries the risk of concealing neck injuries and increased scene time [12]. The practice of prehospital spinal immobilization in HNI may be an overly conservative and overprotective practice for neurological outcomes. More and more studies have focused on this issue and analyzed the subgroup population, such as penetrating HNI, to confirm the hypothesis.

In Wesley B. Vanderlan et al. [15], the authors included penetrating neck trauma, 94% of gunshot injuries, in a level 1 trauma center to evaluate the effect of spinal immobilization on mortality. The result showed that C-spine immobilization in this study was associated with an increased risk of death with an odds ratio of 2.77 (95% CI 1.18–6.49) and an increased risk of cardiopulmonary resuscitation (CPR) with an odds ratio 3.53 (95% CI, 1.06–12.95). In Elliott R. Haut et al. [16], the authors included 45,284 penetrating trauma patients and concluded that spine-immobilized penetrating trauma patients had a higher risk of mortality with an adjusted odds ratio of 2.06 under 95% CI 1.35–3.13 than those who did not undergo prehospital spine immobilization. In both studies, the authors highlighted that the factors that delay c-spinal immobilization in transport may be critically detrimental. In current concepts, the benefit of any prehospital procedure in traumatic injury has been called into question, because their clinical benefit may not be worth delaying definitive care. Moishe Liberman et al. [25] reported that trauma patients who received Advanced Life Support (ALS) did not have better survival rate than whose received Basic Life Support (BLS). In the severe traumatic brain injury population, ALS programs did not improve outcomes and worsened clinical outcomes [26]. A longer prehospital time in traumatic injury may also be associated with an increased risk of poor functional outcomes [27]. Therefore, more studies agree that prehospital cervical and spinal immobilization should be more selective. Our result showed similar data in the HNI population. In the severe injury population, such as consciousness-unclear (GCS ≤ 8), major traumatic injury (ISS ≥ 16 or RTS ≤ 7), and shock status (shock index < 1), the CSI population showed increases in the adjusted OR of mortality greater than that of the nCSI groups, especially in patients with shock status (aOR: 10.103 with 95% CI: 1.673–61.008, *p* = 0.012). We believed that time spent on dealing with significant clinical conditions may provide better clinical outcomes than time spent on spinal immobilization.

There are several limitations to our study. First, our study only reported in-hospital mortality. There was a lack of a functional outcome for C-spinal immobilization. Several studies have also focused on neurological outcomes in trauma patients. A recent PATOS study [28] found that prehospital spinal immobilization was not associated with favorable functional outcomes in trauma patients with spinal injuries, consistent with prior research, such as the work of Mark Hauswald et al. [23], which suggested that prehospital immobilization in cases of blunt spinal injuries has little or no impact on neurological outcomes. Although we believe that the identification of secondary injuries in cases of unstable cervical spine injuries is an ideal endpoint but not a final functional outcome, it is often difficult to distinguish whether sequential neurological deficits represent the progression of the initial traumatic spinal injury or secondary injury resulting from an unstable cervical spine without immobilization, particularly in prehospital care settings. In Taiwan, prehospital transport times are typically under 20 min, with a median transport interval of 7 min and a median prehospital interval of 23 min in Taipei—significantly shorter than in many other countries [29]. Consequently, the identification of secondary injuries related to unstable cervical spine injuries during these brief prehospital periods can be challenging. Patients suspected of head and neck injuries typically receive spinal injury assessments as quickly as possible on arrival in hospitals. Additionally, our hospital’s brain CT scans routinely cover the C1–C2 regions, which are common sites for C-spinal injuries, even when physicians do not explicitly request a C-spinal CT scan. As a result, C-spinal injuries can be promptly identified to minimize the risk of secondary injuries. Therefore, we focused on mortality instead of secondary C-spinal injuries and we believe the incidence of secondary C-spinal injuries is unsignificant.

Second, this study has a retrospective cohort design. The baseline characteristics of the two groups were different, especially in injury severity, which may cause confounding issues through an indication bias. Although a two-arm randomized controlled trial (RCT) is the ideal study design on this issue, the study design may be against research ethics and current practice guidelines. Further, the incidence of secondary injury is low. The included sample number will be large. In 2001, a meta-analysis study focused on this issue and included randomized controlled trials comparing spinal immobilization strategies in trauma patients with suspected spinal cord injuries as selection criteria [30]. The research group showed no randomized controlled trials of spinal immobilization strategies in trauma patients. Up until 2023, there are still no randomized controlled trials on the current issue. Therefore, to minimize the influence of selection bias, we adjusted the differences using univariable, multivariable logistic regression, and subgroup analyses to make our results robust. 

Third, in this database, there is a lack of information regarding neck collar types, such as rigid versus soft collars, the duration of collar wear, rescue airway time, resuscitation time, and the time when spinal injury was definitively excluded. We also did not measure intracranial pressure through parameters like optic nerve sheath diameter or increases in the internal jugular vein. Such data could offer a clearer understanding of the pathophysiological impact of neck collars on mortality.

Furthermore, our study did not distinctly categorize HNI into groups, differentiating between traumatic brain injury, spinal injury, and the combination of traumatic brain injury with spinal injury. In the prehospital setting, emergency medical technicians often make decisions regarding cervical spine immobilization based on the mechanism of injury and the patient’s chief complaints. The definite diagnosis of TBI and spinal cord injury relies on imaging findings, which may not be readily available in the prehospital care context. Lastly, the sample size in our study was small. To obtain more conclusive results, it is essential to conduct large-sample randomized clinical trials in the future.

## 5. Conclusions

Although our results do not support removing a neck collar in all suspected HNI patients, prehospital cervical and spinal immobilization should be more selective in some HNI populations, especially in old age (age ≥ 65), consciousness-unclear (GCS ≤ 8), major traumatic injury (ISS ≥ 16 or RTS ≤ 7), and shock status (shock index ≥ 1). In these populations, the benefit of prehospital C-spinal immobilization may be less. Our study employs a retrospective cohort design, which may introduce selection bias. Therefore, in the future, there is a need for confirmation of our results through a two-arm RCT, as this design is considered ideal for addressing this issue.

## Figures and Tables

**Table 1:** Comparison of demographic characteristics of patients included in the study of in-hospital mortality.

Characteristics	Total Patient	Non-C-Spine Immobilization	C-Spine Immobilization	*p*-Value
Patient number	2733 (100.0%)	1966 (%)	767 (%)	
Age (years)	62 (45–77)	65 (48–79)	54 (37–68)	<0.001
Age < 65	1488 (54.4%)	964 (49.0%)	524 (68.3%)	<0.001
Age ≥ 65	1245 (45.6%)	1002 (51.0%)	243 (31.7%)	
Gender				0.046
Female	1101 (40.3%)	815 (41.5%)	286 (37.3%)	
Male	1632 (59.7%)	1151(58.5%)	481 (62.7%)	
In-hospital vital sign				
SBP	149 (125–173)	150.5 (128–174)	142 (113–168)	<0.001
DBP	85 (73–98)	86 (75–98)	84 (68–98)	<0.001
RR	18 (18–20)	18 (18–20)	18 (18–20)	<0.001
HR	84 (72–96)	84 (74–96)	82 (68–96)	<0.001
Triage GCS	15 (14–15)	15 (15–15)	14 (7–15)	<0.001
Triage GCS ≤ 8	361 (13.2%)	134 (6.8%)	227 (29.6%)	<0.001
Triage				<0.001
1	604 (22.1%)	276 (14.0%)	328 (42.8%)	
2	1499 (54.8%)	1138 (57.9%)	361 (47.1%)	
3	620 (22.7%)	542 (27.6%)	78 (10.2%)	
4 and 5	10 (0.4%)	10 (0.5%)	0 (0%)	
Call trauma team	325 (11.9%)	66 (3.4%)	259 (33.8%)	<0.001
Injury severity				
RTS	7.84 (7.84–7.84)	7.84 (7.84–7.84)	7.84 (5.97–7.84)	<0.001
ISS	9 (8–16)	9 (6–13)	14 (9–25)	<0.001
NISS	10 (8–16)	9 (6–16)	16 (9–27)	<0.001
TRISS	0.97 (0.94–0.99)	0.97 (0.96–0.99)	0.96 (0.67–0.99)	<0.001
ISS ≥ 16	824 (30.2%)	445 (22.6%)	379 (49.4%)	<0.001
RTS ≤ 7	601 (22.0%)	273 (13.9%)	328 (42.8%)	<0.001
TBI				<0.001
Mixed TBI	949 (34.7%)	570 (29.0%)	379 (49.4%)	
Isolated TBI	1784 (65.3%)	1396 (71.0%)	388 (50.6%)	
Prehospital treatment				
Stopping bleeding and banding	869 (31.8%)	441 (22.4%)	428 (55.8%)	<0.001
Spinal board immobilization	537 (19.6%)	53 (2.7%)	484 (63.1%)	<0.001
Splint immobilization	106 (3.9%)	35 (1.8%)	71 (9.3%)	<0.001
Oxygen support	217 (7.9%)	51 (2.6%)	166 (21.6%)	<0.001
Rescue airway ^†^	60 (2.2%)	6 (0.3%)	54 (7.0%)	<0.001
CPR	103 (3.8%)	16 (0.8%)	87 (11.3%)	<0.001
Injury place				<0.001
Home	869 (31.8%)	771 (39.2%)	98 (12.8%)	
Street	1442 (52.8%)	907 (46.1%)	535 (69.8%)	
Work	29 (1.1%)	24 (1.2%)	5 (0.7%)	
Public site	207 (7.6%)	120 (6.1%)	87 (11.3%)	
Others	186 (6.8%)	144 (7.3%)	42 (5.5%)	
Injury type				<0.001
Contusion	931 (34.1%)	845 (43.0%)	86 (11.2%)	
Motor vehicle collision	1153 (42.2%)	683 (34.7%)	470 (61.3%)	
Falling down	457 (16.7%)	295 (15.0%)	162 (21.1%)	
Penetration	22 (0.8%)	20 (1.0%)	2 (0.3%)	
Others	170 (6.2%)	123 (6.3%)	47 (6.1%)	
Past history				<0.001
CNS diseases	226 (8.3%)	191 (9.7%)	35 (4.6%)	
Cardiovascular diseases	825 (30.2%)	694 (35.3%)	131 (17.1%)	
Respiratory diseases	53 (1.9%)	42 (2.1%)	11 (1.4%)	
Gastrointestinal diseases	68 (2.5%)	54 (2.7%)	14 (1.8%)	
Chronic kidney disease	97 (3.5%)	86 (4.4%)	11 (1.4%)	
Diabetes mellitus	350 (12.8%)	296 (15.1%)	54 (7.0%)	
ICU admission	1440 (52.7%)	999 (50.8%)	441 (57.5%)	0.002
Re-admission ICU	29 (1.1%)	19 (1.0%)	10 (1.3%)	0.439
LOS in ICU	4 (3–7)	4 (3–6)	5 (3–9)	<0.001
Operation	651 (23.8%)	430 (21.9%)	221 (28.8%)	<0.001
Re-opertation	103 (3.8%)	59 (3.0%)	44 (5.7%)	0.001
Complications	400 (14.6%)	268 (13.6%)	132 (17.2%)	0.017
Total LOS	7 (4–14)	7 (4–13)	8 (3–17)	0.107
Death	315 (11.5%)	126 (6.4%)	189 (24.6%)	<0.001

Dichotomous and categorical variables are reported as the absolute sample size (percentage); continuous variables are reported as the median (IQR). CPR: cardiopulmonary resuscitation; SBP: systolic blood pressure; DBP: diastolic blood pressure; RR: respiration rate; HR: heart rate; ISS: Injury Severity Score; RTS: Revised Trauma Score; NISS: National Industrial Security System; TRISS: New Trauma and Injury Severity Score; LOS: length of stay; and ICU: intensive care unit. ^†^ Rescue airway includes prehospital supraglottic airway and endotracheal tube insertion.

**Table 2:** Subgroup analysis of spinal immobilization in the consciousness-alert (GCS > 8) and -unclear populations (GCS ≤ 8).

Characteristics	GCS ≤ 8			GCS > 8		
	nCSI	CSI	*p*-Value	nCSI	CSI	*p*-Value
Patient	134	227		1832	540	
Age (years)			0.052			<0.001
Age < 65	79 (59.0%)	157 (69.2%)		885 (48.3%)	367 (68.0%)	
Age ≥ 65	55 (41.0%)	70 (30.8%)		947 (51.7%)	173 (32.0%)	
Gender			1.000			0.101
Female	47 (35.1%)	81 (35.7%)		1064 (58.1%)	335 (62.0%)	
Male	87 (64.9%)	146 (64.3%)		768 (41.9%)	205 (38.0%)	
TBI			<0.001			<0.001
Mixed TBI	47 (35.1%)	130 (57.3%)		523 (28.5%)	249 (46.1%)	
Isolated TBI	87 (64.9%)	97 (42.7%)		1309 (71.5%)	291 (53.9%)	
Injury severity						
ISS ≥ 16	94 (70.1%)	207 (91.2%)	<0.001	351 (19.2%)	172 (31.9%)	<0.001
RTS ≤ 7	134 (100.0%)	227 (100.0%)	-----	139 (7.6%)	101 (18.7%)	<0.001
Call trauma team	29 (21.6%)	167 (73.6%)	<0.001	37 (2.0%)	92 (17.0%)	<0.001
Death	63 (47.0%)	161 (70.9%)	<0.001	63 (3.4%)	28 (5.2%)	0.063

Dichotomous and categorical variables are reported as the absolute sample size (percentage); continuous variables are reported as the median (IQR). nCSI: non-C-spine immobilization; CSI: C-spine immobilization; ISS: Injury Severity Score; RTS: Revised Trauma Score; GCS: Glasgow coma scale; TBI: traumatic brain injury.

**Table 3:** Subgroup analysis of spinal immobilization in isolated TBI and mixed TBI with different consciousness status.

Characteristics	Isolated TBI with GCS ≤ 8			Isolated TBI with GCS > 8		
	nCSI	CSI	*p*-Value	nCSI	CSI	*p*-Value
Patient	87	97		1309	291	
Age (years)			<0.001			<0.001
Age < 65	39 (44.8%)	68 (70.1%)		567 (43.3%)	175 (60.1%)	
Age ≥ 65	48 (55.2%)	29 (29.9%)		742 (56.7%)	116 (39.9%)	
Gender			0.772			0.553
Female	35 (40.2%)	37 (38.1%)		536 (40.9%)	113 (38.8%)	
Male	52 (59.8%)	60 (61.9%)		773 (59.1%)	178 (61.2%)	
Injury severity						
ISS ≥ 16	59 (67.8%)	83 (85.6%)	0.005	253 (19.3%)	79 (27.1%)	0.004
RTS ≤ 7	87 (100.0%)	97 (100.0%)	-----	95 (7.3%)	58 (19.9%)	<0.001
Call trauma team	14 (16.1%)	67 (69.1%)	<0.001	11 (0.8%)	27 (9.3%)	<0.001
Death	44 (50.6%)	59 (60.8%)	0.182	52 (4.0%)	19 (6.5%)	0.060
Characteristics	Mixed TBI with GCS ≤ 8			Mixed TBI with GCS > 8		
	nCSI	CSI	*p*-Value	nCSI	CSI	*p*-Value
Patient	47	130		523	249	
Age (years)			0.035			<0.001
Age < 65	40 (85.1%)	89 (68.5%)		318 (60.8%)	192 (77.1)	
Age ≥ 65	7 (14.9%)	41 (31.5%)		205 (39.2%)	57 (22.9%)	
Gender			0.294			0.051
Female	12 (25.5%)	44 (33.8%)		232 (44.4%)	92 (36.9%)	
Male	35 (74.5%)	86 (66.2%)		291 (55.6%)	157 (63.1%)	
Injury severity						
ISS ≥ 16	35 (74.5%)	124 (95.4%)	<0.001	98 (18.7%)	93 (37.3%)	<0.001
RTS ≤ 7	47 (100.0%)	130 (100.0%)	-----	44 (8.4%)	43 (17.3%)	<0.001
Call trauma team	15 (31.9%)	100 (76.9%)	<0.001	26 (5.0%)	65 (26.1%)	<0.001
Death	19 (40.4%)	102 (78.5%)	<0.001	11 (2.1%)	9 (3.6%)	0.231

Dichotomous and categorical variables are reported as the absolute sample size (percentage); continuous variables are reported as the median (IQR). nCSI: non-C-spine immobilization; CSI: C-spine immobilization; ISS: Injury Severity Score; RTS: Revised Trauma Score; GCS: Glasgow coma scale.

**Table 4:** Multivariable logistic regression of in-hospital mortality.

Variable	Adjusted OR (95% CI)	*p*-Value
Age (years)		
Age < 65	Reference	
Age ≥ 65	2.757 (1.886–4.029)	<0.001
Gender		
Female	Reference	
Male	1.018 (0.0.730–1.419)	0.918
Triage GCS ≤ 8	8.862 (5.425–14.476)	<0.001
ISS ≥ 16	5.380 (3.685–7.853)	<0.001
RTS ≤ 7	2.813 (1.703–4.645)	<0.001
TBI		
Mixed TBI	Reference	
Isolated TBI	0.977 (0.679–1.405)	0.899
Injury type		
Contusion	Reference	
Motor vehicle collision	0.671 (0.418–1.078)	0.099
Falling down	0.907 (0.551–1.493)	0.700
Penetration	0.833 (0.114–6.091)	0.857
Others	1.543 (0.800–2.976)	0.196
Call trauma team	1.168 (0.753–1.810)	0.488
Neck collar immobilization	1.850 (1.240–2.760)	0.003

**Table 5:** Multivariable logistic regression of in-hospital mortality in subgroups.

Subgroups	Non-C-Spine Immobilization	C-Spine Immobilization	*p*-Value
	Adjusted OR (95% CI)	Adjusted OR (95% CI)	
Age			
Age < 65	Reference	1.708 (0.981–2.976)	0.059
Age ≥ 65	Reference	2.035 (1.108–3.736)	0.022
GCS			
GCS ≤ 8	Reference	2.495 (1.342–4.640)	0.004
GCS > 8	Reference	1.434 (0.788–2.610)	0.238
ISS			
ISS < 16	Reference	1.084 (0.457–2.572)	0.855
ISS ≥ 16	Reference	2.248 (1.388–3.642)	0.001
RTS			
RTS ≤ 7	Reference	2.273 (1.383–3.733)	0.001
RTS > 7	Reference	1.250 (0.576–2.715)	0.573
TBI type			
Mixed TBI	Reference	2.181 (1.079–4.407)	0.030
Isolated TBI	Reference	1.685 (1.019–2.785)	0.042
Activation of trauma team			
Call trauma team	Reference	2.135 (0.854–5.338)	0.105
Non-call trauma team	Reference	1.942 (1.230–3.067)	0.004
Shock status			
Shock index ≥ 1	Reference	10.103 (1.673–61.008)	0.012
Shock index < 1	Reference	1.469 (0.897–2.407)	0.126

Co-variables used in multivariable logistic regression included age, sex, mechanism of injury, type of traumatic brain injury, Injury Severity Score, and Revised Trauma Score, except the variable of the subgroup. ISS: Injury Severity Score; RTS: Revised Trauma Score; GCS: Glasgow coma scale; TBI: traumatic brain injury.

## Data Availability

Data are contained within the article.
